# Trace element zinc and skin disorders

**DOI:** 10.3389/fmed.2022.1093868

**Published:** 2023-01-17

**Authors:** Pan Zou, Yuxin Du, Chunguang Yang, Yuchun Cao

**Affiliations:** ^1^Department of Dermatology, Tongji Hospital, Tongji Medical College, Huazhong University of Science and Technology, Wuhan, China; ^2^Department of Urology, Tongji Hospital, Tongji Medical College, Huazhong University of Science and Technology, Wuhan, China

**Keywords:** zinc, skin, treatment, infections, inflammation

## Abstract

Zinc is a necessary trace element and an important constituent of proteins and other biological molecules. It has many biological functions, including antioxidant, skin and mucous membrane integrity maintenance, and the promotion of various enzymatic and transcriptional responses. The skin contains the third most zinc in the organism. Zinc deficiency can lead to a range of skin diseases. Except for acrodermatitis enteropathic, a rare genetic zinc deficiency, it has also been reported in other diseases. In recent years, zinc supplementation has been widely used for various skin conditions, including infectious diseases (viral warts, genital herpes, cutaneous leishmaniasis, leprosy), inflammatory diseases (hidradenitis suppurativa, acne vulgaris, rosacea, eczematous dermatitis, seborrheic dermatitis, psoriasis, Behcet's disease, oral lichen planus), pigmentary diseases (vitiligo, melasma), tumor-associated diseases (basal cell carcinoma), endocrine and metabolic diseases (necrolytic migratory erythema, necrolytic acral erythema), hair diseases (alopecia), and so on. We reviewed the literature on zinc application in dermatology to provide references for better use.

## 1. Introduction

Zinc is a necessary trace element for humans and essential in various vital movements. Zinc is present in more than 300 enzymes closely related to metabolism, in which zinc acts on catalysis, structure formation, and activity regulation. In addition, zinc is also abundant in nucleic acid-binding proteins, which control the growth and development of the body. Zinc finger proteins are the most typical and crucial of them.

Zinc is mainly absorbed by the small intestine, then enters the blood circulation and is delivered to various tissues. Finally, zinc is excreted by feces, urine, and exfoliation of the epidermis. Zinc is mainly distributed in cells and concentrated in muscle (60%), bone (30%), and skin (5%) (1). Zinc transporters regulate zinc levels. Zinc transporters include the SLC39A/ZIP family and the SLC30A/ZnT family: the SLC39A family transports zinc ions into the cell, and the SLC30A family is responsible for transporting zinc ions out of the cell. Zinc transporters are significant in the maintenance of zinc homeostasis and assume a variety of physiological functions. For example, ZIP10 controls the formation of epidermis and hair follicles, and ZIP4 regulates dietary zinc absorption. Zinc transported by zinc transporters can be regarded as a “zinc signal” to regulate the activities of organelles and subsequently participate in specific physiological activities. Both zinc deficiency and zinc excess cause “zinc stress,” which impairs the expression of zinc transporters and zinc signal transduction, eventually leading to pathophysiological processes related to diseases (2, 3).

Zinc has several physiological effects. Sufficient zinc can preserve the completeness of the tissue barriers and prevent pathogen infection. Zinc is involved in immunomodulation and is assumed to be decisive in activating pro- vs. anti-inflammatory genes. Zinc is indispensable for hematopoietic function and has antiandrogen effects (4, 5). Zinc excess can cause poisoning, with symptoms such as gastroenteritis, gastrointestinal bleeding, microcytosis, relative neutropenia, hypoceruloplasminemia, and in prolonged cases, anemia secondary to Cu deficiency (3). Zinc deficiency can lead to various abnormalities, such as pathogen infection, immune dysregulation, and inflammatory activation. The proportion of people with zinc deficiency varies widely across countries, with ~2 billion globally affected by zinc deficiency (6). Insufficient intake, malabsorption, and excessive zinc loss can cause zinc deficiency. In developing countries, the primary factor leading to zinc deficiency is the high intake of phytate grain protein (7).

Skin is the third most zinc-containing organ, and zinc deficiency is concerned with multiple skin diseases (1). Zinc has a prolonged historical use in the management of skin diseases. Zinc oxide can be used as a sunscreen to prevent skin diseases associated with sunlight exposure (8). Oral zinc in treating acrodermatitis enteropathies is also well-known (9). In recent years, more and more clinical trials have been conducted to treat various types of skin disorders, like acne, viral warts, and cutaneous ulcers. Some of the trials have achieved remarkable results (Table 1). However, the relationship between zinc and etiology and pathogenesis still needs to be fully understood (10, 34, 53).

**Table 1:** Human studies of zinc supplementation for the treatment of skin disorders.

**Disease**	**Type**	**Intervention**	**Control or placebo**	**No**.	**Results**	**Side effect**	**Reference**
Viral warts	Placebo-controlled clinical trial	Oral zinc sulfate 10 mg/kg/day for 2 mo	Placebo	43	The overall response was complete clearance of warts observed in 20 patients (86·9%) after 2 mo; No patient of the placebo-treated group showed any response	Nausea, vomiting and mild epigastric pain	(10)
	Randomized clinical trial	(1) Intralesional 2% zinc sulfate solution once every 2 weeks for 2 mo (2) Intralesional 2% vitamin D3	Normal saline	105	Comparing the zinc group and the saline group as regards the response to treatment, no statistically significant difference after the first session, while a statistically significant difference after the second, third, and the fourth session; no statistically significant difference between the zinc group and vitamin D3 group	Severe pain	(11)
Epidermodysplasia verruciformis	Case study	Oral zinc sulfate 10 mg/kg/day for 3 mo	-	1	Effective	None	(12)
Genital herpes	Randomized clinical trial	Topical 1, 2 and 4% zinc sulfate for 3 mo	-	90	4% zinc sulfate was the most efficacious	None	(13)
Leprosy	-	Oral zinc sulfate 220 mg/day for 4 mo	-	40	Improvement in the frequency, duration and severity of reactions; reduction in steroid requirement	-	(14)
Cutaneous leishmaniasis	Randomized clinical trial	(1) Intralesional 2% zinc sulfate solution once/10–15day (2) Intralesional 7% sodium chloride solutions (3) Sodium stibogluconate	Control	63	Comparable cure rates of the three treatments; however zinc sulfate gave a high cure rate (94.8%)	Pain	(15)
	Prospective, double-blind, case-control clinical trial	Intralesional 2% zinc sulfate solution every 2 weeks	Glucantime	66	The cure rates were 60% for glucantime and 83.8% for zinc sulfate. After the second and fourth weeks, the efficacy of treatment with ZS was higher than that with MA (*p* < 0.01), but after 6 weeks no significant differences between the two groups	Severe pain, vasovagal shock	(16)
	Randomized, double-blind, clinical trial	Intralesional 2% zinc sulfate solution	Glucantime	72	Less effective than glucantime	-	(17)
	Randomized clinical trial	Intralesional 2% zinc sulfate solution once every 2 weeks	Glucantime	45	Less effective than glucantime	Pain, hyperpigmentation, burning, necrosis	(18)
Hidradenitis suppurativa	Pilot study	Oral zinc gluconate 90 mg/day for 4 mo	-	22	A clinical response in all patients, with 8 CR and 14 PR	Nausea, vomiting, abdominal distension, oesophagitis	(19)
	Retrospective study	Oral zinc gluconate 90 mg/day and topical 2% triclosan bid for 3 mo	-	54	The median mHSS significantly decreased (*p* < 0.0001); The mean ± SD number of inflammatory nodules per patient significantly decreased	Nausea, abdominal pain	(20)
	Retrospective study	Oral zinc gluconate 90 mg/day and nicotinamide 30 mg/day for 90 days	Control	92	Significant reduction in the number and mean duration of acute flares; a marked reduction in mean VAS, DLQI, and International HS SSS scores compared with the control group	Nausea	(21)
Rosacea	Randomized, double-blind, clinical trial	Oral zinc sulfate 100 mg tid	Placebo capsules	19	The disease severity score started to decrease directly after the first month of therapy with zinc sulfate to a significantly lower level; while high in the first 3 months of therapy on placebo	Mild gastric upset	(22)
	Single-blinded, comparative trial	Topical 5% zinc sulfate solution bid for 2 mo	2% tea lotion bid	22	Significant in reducing the disease severity score in acne rosacea	Erythema, itching, burning, eye involvement, gastrointestinal problem	(23)
	Randomized, double-blind clinical trial	Oral zinc sulfate 220 mg bid for 3 mo	Placebo	53	No differences in reducing the rosacea severity scores between placebo and zinc group	Nausea, discomfort, gas, diarrhea, constipation	(24)
Atopic dermatitis	Uncontrolled pilot trial	ZnO textile in the form of trousers and long-sleeve shirts	-	12	Pruritus, sleep quality, and clinical cutaneous symptoms improved	-	(25)
Diaper dermatitis	Prospective, open-label trial	A zinc gluconate-taurine/zinc oxide and panthenol/ glycerin/ Butyrospermum parkii butter barrier cream bid for 30 days	-	20	At 30 days, a significant decrease of CEA severity comparing clinical scores at baseline	None	(26)
Seborrhoeic dermatitis	Open randomized, parallel-group trial	1% zinc pyrithione twice weekly for 4 wk	2% ketoconazole twice weekly for 4 wk	331	(1) Significantly greater improvement in TDSS in the control group (2) Significantly more numerous of relapse (*p* < 0.03) in the zinc group than in the contral group	Erythema (*n* = 2)	(27)
	Open-label study	An herbal and zinc pyrithione shampoo and a scalp lotion at least twice weekly	-	50	TDSS improved by 65 percent after six weeks of treatment (*p* < 0.05 as compared with baseline)	None	(28)
Psoriasis	Randomized double-blind clinical trial	Topical 0.25% zinc pyrithione in an emollient	Placebo	60	Effective in decreasing the severity of indurations, erythema, scaling, and PASI scores	None	(29)
	(1) A double-blind cross-over trial (2) An open trial	(1) Oral zinc sulfate 220 mg tid for 6 wk (2) Oral zinc sulfate 220 mg tid for 6 mo	(1) Placebo (2) -	(1) 24 (2) 11	(1) The joint pains were significantly reduced (*P* < 0.02) in all assessments (overall condition, morning stiffness, joint pains and functional capacity of the joints (2) A significant reduction of joint pains, the morning stiffness (*P* < 0.05) and an improvement of the overall condition (*P* < 0.01)	Slight dyspepsia (nausea, epigastric and diffuse abdominal dull pains, loose bowels, constipation)	(30)
Oral lichen planus	Case study	Oral zinc acetate 50 mg bid for 8 wk combined with 0.1% triamcinolone acetonide oral paste for 1 wk and tablet ascazin 50 mg continued for 8 wk	None	3	(1) The VAS decreased from 10/10 to 1/10 in a span of 8 weeks (2) There were 80% 90% decreases in size of the lesions (3) No recurrence of lesions or burning sensation at follow up visits till 2 months after stopping treatment.	-	(31)
	Single-centered, single-blinded, prospective, double-arm trial	Oral zinc acetate 50 mg bid for 8 wk combined with 0.1% triamcinolone acetonide orabase for 1 wk	0.1% triamcinolone acetonide orabase bid	40	(1) Both groups gradual decrease burning sensation (2) Gradual decrease in the size of the lesion from the first visit till the follow-up period which was statistically significant in both the groups (*P* = 0.000)	None	(32)
Behcet's disease	Randomized, controlled, double-blind trial	Oral zinc sulfate tablets 100 mg tid for 3 mo and then crossed over to placeco	Placebo tablet tid and then crossed over to zinc sulfate	27	(1) The serum zinc levels rised after treatment with zinc sulfate (2) Both CMI and ESR were significant inverse correlated with serum zinc level. All values of Hb concentration and total WBC count were normal before therapy. No significant changes were seen in Hb and WBC count.	None	(33)
Ulcers and wounds	Randomized double-blind placebo-controlled clinical trial	Oral zinc sulfate 220 mg qd for 12 wk	Placebo	60	(1) A significant rise in serum zinc levels (2) Significant reductions in ulcer length	-	(34)
	Randomized controlled clinical tiral	Calcium alginate dressings with ZnO nanoparticles once/48 h for10 wk	Calcium alginate dressings without ZnO nanoparticles	26	Healing of the experimental and control groups, respectively was statistically significant (*p* = 0.011)	None	(35)
Necrolytic migratory erythema	Case study	Oral zinc sulfate 220 mg tid, amino acid infusions and ntralesional octreotide acetate 20 mg	-	1	Improved and healed completely in 1 month with residual pigmentary changes and scarring	-	(36)
Necrolytic acral erythema	Case study	Oral zinc sulfate 220 mg bid	-	1	Complete resolution of symptoms, exacerbation of signs and symptoms when oral zinc sulfate	-	(37)
	Case study	Oral zinc sulfate 220 mg bid for 3 mo	-	5	Good responsed (*n* = 2) Moderate response (*n* = 3)	Jaundice	(38)
	Case study	Zinc oxide ointment	-	1	(1) Serum zinc level improved (2) Improved spontaneously with complete healing at 2 months	-	(39)
Alopecias	Control group study	Oral zinc aspartate 100 mg, biotin 20 mg, and topical 0.25% clobetasol propionate qd	Deflazacort 1 mg/kd/day for 20 days, then every week the dose reduced by 5 mg until a maintenance dose of 5 mg/day for 1 y	18	(1) A better outcome in those on the protocol treatment (2) No statistical differences between the treatments in terms of eyebrow regrowth	None	(40)
	Randomized, investigator-blinded, parallel-group clinical study	(1) 1% pyrithione zinc shampoo qd (2) Topical 5% minoxidil solution + placebo (3) Combination of the 1% pyrithione zinc shampoo and the 5% minoxidil	A placebo	200	Hair counts Significant (*P* three groups relative to the placebo shampoo after 9 wk, 1% pyrithione zinc shampoo was slightly less than half that for the minoxidil topical solution	-	(41)
	-	Oral zinc gluconate 50 mg/day for 12 wk	-	15	(1) The serum zinc level increased significantly (2) No statistically significant hair regrowth	-	(42)
	Randomized, control trial	Zinc and arginine lotion	Placebo	40	Number of hairs by Pull tests significantly decreased	-	(43)
Vitiligo	Randomized clinical trial	Oral zinc sulfate 220 mg bid (10 mg/kg for children) with topical corticosteroid	Topical corticosteroid bid	35	No statistically significant difference between the two group	Tolerable gastric burning (*n* = 2)	(44)
Melasma	-	Topical 10% zinc sulfate solution bid for 2 mo	-	14	The mean MASI score decreased from 9.45 to 4.70, it was statistically significant (*p* < 0.0005)	Mild stinging sensation	(45)
	Investigator-blinded, randomized, control trial study	Topical 10% zinc sulfate solution bid	4% hydroquinone cream bid	70	Reduction of the MASI score in both groups, however, the reduction in control group was more significant	Mild burning and erythema	(46)
	Randomized, double-blind, controlled tria	Topical 10% zinc sulfate solution qd for 2 mo	4% hydroquinone solutions qd	93	The MASI score fell significantly in both groups, but a greater decrease in the control group	Mild postinflammatory pigmentation	(47)
Basal cell carcinoma	Open-label case interventional study	Intralesional 2% zinc gluconate solution once every 2 wk in 30 lesions	Intralesional 4% zinc sulfate solution and 2% xylocaine in 70 lesions	100 lesions	All lesions showed clinical cure	Pain, swelling, erythema	(48)
Actinic keratosis	Single-blind trial	Topical 25% zinc sulfate bid for 12 wk	None	18	6 patients showed statistically significant good response, 8 patients showed statistically significant partial response after 10 wk	Mild transient burning sensation (*n* = 6)	(49)
Hailey-hailey disease	Case study	Topical 50% zinc oxide paste alone on his left axilla bid	Topical 50% zinc oxide paste with 0.1% zinc oxide paste bid on his right axilla	1	The axilla treated with zinc oxide paste alone had a better outcome	None	(50)
Erosive pustular dermatosis	Retrospective study	Topical 25% powdered zinc oxide two or three times a week for 12 wk after using TCs for 10 days	TCs two or three times a week for 12 wk	51	(1) Effective in all patients (2) The TC-only group had more relapses compared with the TC + topical zinc oxide group	-	(51)
	Case study	Topical 25% zinc oxide bid	-	1	Complete healing achieved in 4 weeks, no relapses during the 6-month follow-up,	-	(52)

mo, month; CR, complete remissions; PR, partial remissions; bid, twice daily; mHSS, modified HS score; VAS, visual analog scale; DLQI, dermatology life quality index; SSS, severity score system; tid, three times daily; CEA, clinical erythema assessment; wk, week; qd, once daily; h, hour; CMI, clinical manifestations index; TDSS, total dandruff severity score; MASI, melasma area and severity index; TCs, topical corticosteroids.

## 2. Infectious diseases

### 2.1. Viral warts

Viral warts are a type of skin disorder resulting from human papillomavirus (HPV) infection and clinically manifest as benign skin neoplasms, including verruca plana, verruca vulgaris, and condyloma acuminatum. Currently, the main therapeutic choice is medication such as salicylic acid, urea, etc. However, zinc preparations have been found to be effective as a treatment modality. Korkmaz et al. (54) found that zinc levels were significantly lower in the verruca vulgaris group than in healthy volunteers(77.73 vs. 91.50 μg/dL, *P* = 0.002). The study of Luong et al. (55) suggested that the severity of genital warts may be related to the patient's low serum zinc levels. Therefore, we hypothesized that there was a particular relationship between zinc deficiency and the pathogenesis of viral warts. The immunomodulating effects of zinc can resist virus exposure by affecting cytokine synthesis. Zinc deficiency reduces the lytic activity of natural killer cells, damages natural killer T cell cytotoxicity and immune signaling, affects the neuroendocrine-immune pathway, and adjusts cytokine production in mast cells. Furthermore, adaptive immune system activity depends on zinc, especially T lymphocytes. Without zinc, the functions of T helper type 1, the lymphocyte responsible for antivirus operation, deteriorate. Moreover, cytotoxic T lymphocytes depend heavily on zinc to clear viral infections (56). Zinc deficiency reduces antiviral immunity, and zinc supplementation can help treat viral warts. 86.9% of patients with viral warts (common, plantar, and plane) had complete resolution after 2 months of oral administration of zinc sulfate in a randomized controlled clinical study. However, there was no response in the placebo group (10). According to El Sayed et al. (11), 105 patients with plantar warts participated in the three-armed randomized clinical trial. On average, patients were divided into three groups, each receiving intralesional 2% zinc sulfate, 2% vitamin D, or physiological saline. Ultimately, more patients in the zinc group (71.4%) and vitamin D3 group (62.9%) than in the saline group (40%) had complete remission. The most apparent adverse reaction was pain while injecting zinc sulfate. A multicenter, randomized clinical trial compared an external nitrizinc complex solution with cryotherapy for treating anogenital warts. The results showed that both were effective for small (<5 mm) external anogenital warts. Nitric complex solution was better tolerated and had a lower recurrence rate (57).

### 2.2. Epidermodysplasia verruciformis

Epidermodysplasia verruciformis (EV) is characterized by widespread flat warts and wart-like lesions. The disease is genetically susceptible to HPVs. Most epidermodysplasia verruciformis cases resulted from invalidating mutations in the EVER1 or EVER2 gene. A complex formed by EVER1 and EVER2 proteins interacts with zinc transporter one (ZNT1). EVER and ZNT1 proteins affect cellular zinc allocation. EVER2 suppressed the entrance of free zinc into the nucleolus (58). This may be a breakthrough in the treatment of EVs with zinc. Sharma et al. (12) reported an EV patient with squamous cell carcinoma. The patient received oral zinc sulfate 550 mg daily for 12 weeks. The follow-up visit lasted for 6 months. The primary outcome of the measurement was complete verrucae removal.

### 2.3. Genital herpes

Genital herpes is a sexually transmitted disease caused by herpes simplex virus−1(HSV-1) or herpes simplex virus-2(HSV-2) infection. It's the primary cause of genital ulcers and is chronic, recurrent, and refractory. José et al. (59) found that zinc acetate/carrageenan gels were revealed to prevent mice from high-dose (10^6^ PFU) HSV-2 vaginal or rectal challenge, and carrageenan had activity against HSV-2 *in vitro*. Mahajan reported that zinc sulfate was an effective therapeutic modality in pain, tingling, burning sensation, and prolonged remission. Moreover, topical 4% ZnSO4 was the most resultful out of the three concentrations (1, 2, and 4%), without any side effects (13). A type of specially designed zinc oxide tetrapod nanoparticle was reported to be useful as a novel immunoprotective agent against genital herpes (60). In the future, we can expect more clinical trials to provide evidence.

### 2.4. Leprosy

Leprosy, resulting from Mycobacterium leprae infection, mainly influences humans' skin and peripheral nerves. A case-control trial including 100 leprosy patients (50 multibacillary and 50 paucibacillary) and 100 controls examined zinc, vitamin C, and selenium serum levels. A decrease in serum zinc levels was measured in patients with leprosy (paucibacillary leprosy: 89.86 ± 20.712 μg/dL, multibacillary leprosy: 81.41 ± 18.61 μg/dL, control group: 107.34 ± 3.98 μg/dL), it might present prior to the disease onset due to malnutrition which may have accelerated the development of leprosy. It also might be result from disease pathogenesis or related to the antioxidant based treatment (61). Reinar et al. (62) summarized three studies of ulcerations caused by nerve damage in leprosy that compared zinc tape with other interventions (magnesium sulfate, povidone-iodine, gauze soaked) and reported results in favor of zinc tape. Forty patients with chronic recurrent nodular leprosy were given oral zinc sulfate for 4 months. The frequency, duration, and severity of reactions were markedly improved after zinc therapy (14).

### 2.5. Cutaneous leishmaniasis

Leishmaniasis is a zoonotic disease due to the parasite Leishmania transmitted between arthropods and mammals. As an essential trace element, zinc is crucial to develop and activate immune cell function. Several studies have shown reduced zinc levels in leishmaniasis patients (98.58 ± 19.7 vs. 126.38 ± 40.2 μg/dL, *P* < 0.001) (63–65). Zinc deficiency may cause an imbalance of Th1 and Th2 cells and decrease cytotoxic T cell frequency. Susceptible mice treated with zinc sulfate could recruit protective T cells and conduct a Th1-type immune response (66). Pentavalent antimonials are the first-line treatment used extensively worldwide against cutaneous leishmaniasis (CL). However, this treatment is expensive and causes severe adverse reactions (67). The effects of zinc in treating CL have been examined. Nevertheless, the results were not the same. In an experimental comparison of intralesional injection of 2% ZnSO_4_, 7% NaCl solution, or sodium stibogluconate, a single injection of zinc sulfate had a high recovery rate (94.8%) (15). Iraji et al. (16) observed comparable response rates between intralesional 2% zinc sulfate and meglumine antimoniate. However, Firooz et al. (17) and Maleki et al. (18) found that intralesional 2% ZnSO_4_ was less practical than the meglumine antimoniate. All of these experiments had relatively small subjects. More extensive randomized trials are needed to confirm zinc as an adjunctive treatment for leishmaniasis.

## 3. Inflammatory diseases

### 3.1. Hidradenitis suppurativa

Hidradenitis suppurativa (HS) is a chronic disease characterized by recurrent, painful, deep round nodules and abscesses on the skin containing apocrine glands. Its main features are festering subterranean passage and hypertrophic scarring (68). Brocard et al. (19) tested 22 patients with HS who received zinc gluconate (90 mg/day for 4 months), with complete remissions in 8 patients and partial remissions in 14 patients. After that, Hurley's grade I patients required an average of 43.5 mg of zinc gluconate daily, and Hurley's grade II patients required an average of 71 mg daily to prevent disease recurrence. This is also the first report on zinc efficiency in HS. Hessam et al. (20) analyzed 54 patients treated with oral zinc gluconate and topical triclosan. They found that the treatment protocol was effective at treating inflammatory lesions (such as inflammatory nodules, papules, or pustules) but had little effect on treating chronic lesions (such as tubules, interconnecting sinuses, and hypertrophic scars). Offidani et al. (69) analyzed the condition of 8 prepubertal HS patients. They concluded that short-term control of HS flares by azithromycin and oral zinc as routine maintenance therapy was considered a good plan. Molinelli et al. (21) then performed a controlled retrospective clinical study on 92 patients with mild-to-moderate HS. Oral zinc gluconate and nicotinamide were validated in the maintenance phase of them. There was no obvious sign of relapse after drug withdrawal. Zinc gluconate has an anti-inflammatory or proinflammatory activity that may explain the effectiveness of the treatment. The main adverse reactions were abdominal pain, nausea, and other gastrointestinal symptoms. Nevertheless, Stashower et al. (70) reported copper-deficiency anemia resulting from supplemental zinc therapy for HS. Therefore, we should be concerned about this potentially profound adverse reaction when using zinc administration as an adjunct strategy.

### 3.2. Acne vulgaris

Acne vulgaris is the most common chronic inflammatory hair follicle sebaceous gland disease in dermatology, which tends to occur in adolescent males and females. Its pathogenesis is mainly related to sex hormone levels, sebum secretion, Propionibacterium acnes proliferation, and hair follicle sebaceous duct (71). One of the characteristics leading to the development of an acne lesion is hypercornification of the follicle wall. Immune-mediated inflammatory processes might involve CD4+ lymphocytes and macrophages stimulating the pilosebaceous vasculature to precede follicular hyperkeratinization. Zinc has antibacterial and anti-inflammatory effects and may reduce sebum secretion. It is also often used in dermatology as an adjunctive treatment for acne. A recent meta-analysis involving 25 articles and 2,445 subjects confirmed low serum zinc levels in patients with acne (96.308 ± 4.053 vs. 102.442 ± 3.744 μg/dL, 95% CI −24.098– −0.486, *P* = 0.041). Zinc is effective in treating acne, especially in reducing the number of inflammatory episodes. There was no statistically significant difference in the incidence of adverse reactions like nausea, vomiting, or bellyache between oral or topical zinc with other methods (placebo, minocycline, clindamycin, erythromycin, etc.) (72). Cervantes et al. (73) also made a detailed comparison, including 32 articles in their review. Up to 12 studies examined the effectiveness of zinc as a unitary active ingredient, eight of which showed positive. Six studies combined zinc with other compounds and found it more effective in reducing acne damage (acne severity grade, papule and pustule count, comedone count, inflammatory lesion count, propionibacterium count, Free fatty acid on skin surface count, nodule and open comedones count). Different results were revealed in trials that compared zinc-containing compounds with other strategies of acne therapy. Different doses and uses of zinc have been reported in various trials, and the best treatment for acne vulgaris needs to be evaluated in more extensive, double-blind controlled studies.

### 3.3. Rosacea

Rosacea is a chronic and recurrent inflammatory skin disorder mainly influencing the central areas of the face (74). Antibiotics, immunosuppressants, retinoids, and vascular lasers are commonly used. Sharquie et al. (22) conducted a double-blind, randomized controlled trial of zinc sulfate 100 mg three times daily for rosacea in 25 patients. After 3 months of treatment, a statistically significant reduction in post-study disease activity without serious side effects was observed in 19 patients. A subsequent randomized, single-blind in 22 patients by Sharquie et al. (23) compared the efficacy of a 5% zinc sulfate solution with 2% tea lotion in the treatment of rosacea. They found that a 5% zinc sulfate solution significantly reduced the disease severity score in acne rosacea. However, Bamford et al. (24) and Gessert et al. (75) compared zinc sulfate with placebo for rosacea therapy. The results showed that oral zinc sulfate did not significantly improve rosacea severity compared with a placebo and was even less effective than a placebo in these studies.

### 3.4. Eczematous dermatitis

Eczematous dermatitis comprises a diverse group of skin diseases. Papular vesicles and exudation were the main manifestations in the acute phase, while lichenoid degeneration was the primary manifestation in the chronic phase. Except for classic contact dermatitis and atopic dermatitis (AD), there is a group of inflammatory skin diseases that share both the main features of eczema and unique etiologic features, such as asteatotic eczema, auto-sensitization dermatitis, stasis dermatitis, and diaper dermatitis. Although studies have shown no convincing evidence to support that oral zinc supplementation improves eczemas (76), topical zinc can be used in the supportive treatment of eczemas due to its anti-inflammatory properties and increased reepithelialization. Marit et al. (77) found that topical application of ZnO nanoparticles inhibited allergen-induced skin inflammation but induced potent IgE production in a mouse model of AD. It might be caused by non-specific reactions of released Zn2+ ions affecting the IgE production capabilities of the B-cells. Cornelia et al. (25) observed that AD patients who wore zinc oxide-impregnated fabrics had significant improvements in disease severity, pruritus, and sleep quality compared with controls. Zinc oxide is also an effective adjunctive treatment for diaper dermatitis, although it is not as effective as topical glucocorticoids (7, 26).

### 3.5. Seborrhoeic dermatitis

Seborrhoeic dermatitis (SD) is an inflammatory skin disease commonly seen in the head, face, chest, and back with more sebum, accompanied by erythema, scales, and pruritus. The pathogenesis of seborrheic dermatitis is related to Malassezia colonization, immune response, androgens, and skin barrier damage. A case-control study found that SD patients had significantly lower serum zinc levels than controls (79.16 ±12.17 vs. 84.88 ± 13.59 μg/dL, respectively; *P* = 0.045). Zinc may be involved in disease pathogenesis through multiple mechanisms, such as inflammatory processes, epithelial differentiation, antifungal properties, and antiandrogenic effects (78). Zinc pyrithione (ZPT) 1% is an active ingredient in dandruff shampoos, and shampoos containing it are also an effective treatment for scalp seborrheic dermatitis. Studies have shown that ZPT in shampoo can significantly reduce inflammation and scale, but its therapeutic response is not as good as 1% ketoconazole (27). Barak-Shinar et al. (28) demonstrated that using an herbal and zinc pyrithione-based shampoo and scalp lotion improved the symptoms and severity of scalp SD and dandruff symptoms. Topical zinc is used in treating SD based on its cytotoxic effect on fungi, antiandrogenic effect, and antiproliferative effect.

### 3.6. Psoriasis and psoriatic arthritis

Psoriasis, an immune-mediated polygenic genetic disease, can be induced by environmental factors. Approximately 20–30% of patients with psoriasis have comorbid arthritis or develop psoriatic arthritis in the future, dramatically affecting the patient's quality of life. Jyoti et al. (79) found that serum zinc analysis revealed a non-significant difference between the psoriatic and control groups (mild disease: 99.81 ± 20.80 μg/dL, severe disease: 103.63 ± 24.90 μg/dL, control group: 97.87 ± 15.54 μg/dL, *P* = 0.505). Effective treatment methods have been the focus of research based on psoriasis' chronic and recurrent characteristics. The topical formulation of zinc pyrithione was reported for treating localized psoriasis (area < 10%) (29). However, oral zinc supplements' effectiveness in treating psoriasis is controversial. Clemmensen et al. (30) found that oral zinc sulfate was effective in 24 patients with psoriatic arthritis without any severe side effects. In contrast, Burrows et al. (80) reported that oral zinc sulfate treatment of plaque psoriasis did not produce clinically significant improvement. The efficacy and safety of oral zinc in treating psoriasis and psoriatic arthritis require more formal and extensive sample-size studies.

### 3.7. Oral lichen planus

Oral lichen planus (OLP) is an autoimmune disease mediated by T cells which are considered a potentially malignant disorder with a rate of transformation to oral cancer varying between 0.5 and 2% (31). The etiology and pathogenesis of OLP are still unclear, and there is no specific treatment. Most OLP patients are treated with comprehensive treatment, including topical drugs, glucocorticoids, and immunosuppressants. A preliminary study found a decrease in serum zinc levels of OLP patients in contrast with controls (14.37 ± 3.64 vs. 16.47 ± 2.10 μmol/L, *P* < 0.001) (81). Moreover, serum zinc levels decreased significantly in the erosive lichen planus group than in the non-erosive lichen planus group (8.3 ± 1.15 vs. 11.15 ± 0.92 μg/dL, *P* < 0.05). This discovery may demonstrate zinc's promising function in developing OLP (82). Case reports showed that oral zinc acetate (50 mg) improved lesional size and global index scores in symptomatic OLP (31). Chantada et al. (32) found that 50 mg oral zinc and 0.1% triamcinolone orabase were more effective than 0.1% triamcinolone orabase in improving burning sensation and lesional size. The therapeutic effect of zinc on OLP requires further high-quality research.

### 3.8. Behcet's disease

Behcet's disease (BD) often presents with recurrent oral ulcers, genital ulcers, or eye lesions. Its etiology may be related to infection, autoimmunity, and heredity. A double-blind crossover study showed that the mean serum zinc level in BD patients was significantly lower than the mean serum zinc levels in the healthy control group (67.56 ± 3.3 vs. 85 ± 2.8 μg/dL, *P* < 0.05). Oral administration of zinc sulfate increased serum zinc levels in patients, which were negatively correlated with the clinical manifestations index of BD (33). Amir et al. (83) found that zinc supplementation significantly improved non-ocular BD activity and Toll-like receptor (TLR-2) expression in BD patients. TLR-2 is involved in the pathogenesis of BD.

### 3.9. Ulcers and wounds

Zinc is a co-factor of various metalloproteins necessary for cell proliferation, growth, cell membrane recovery, and immune system functions. It plays a crucial role in wound healing (84). Various causes can cause ulcers on the skin, including diabetic foot ulcers (DFUs), pressure ulcers, sickle cell leg ulcers (SCLUs), and arterial and venous leg ulcers. A study was conducted in Mexico to understand the frequency of hypozincemia in DFU and its association with metabolic and clinical features (85). The results showed that the incidence of hypozincemia in diabetic DFU patients was higher, which was a risk factor for DFU (OR = 5.2, 95% CI 2.139–12.65, *p* = 0.0004). Momen-Heravi et al. (34) conducted a randomized, double-blind, placebo-controlled trial to determine the effects of zinc supplementation on wound healing and metabolic status in patients with DFU. They concluded that zinc supplementation for 12 weeks in DFUs had a beneficial effect on parameters of ulcer size and metabolic characteristics. However, another study found no difference between calcium alginate dressings with ZnO NPs (experimental group) and calcium alginate alone (control group) in treating DFU (35). A recent systematic review and meta-analysis found seven relevant articles examining the use of zinc in pressure ulcers (86). The study confirmed that zinc treatment could promote wound healing and suggested that healthcare providers provide zinc to patients during pressure ulcer therapy. Serjeant et al. (87) performed a randomized placebo-controlled trial and observed that SCLU healed faster in the zinc supplement group, and this is the only current study of zinc in the treatment of SCLU. Wilkinson et al. (88) conducted a new evaluation of the efficacy of oral zinc in treating arterial and venous ulcers of the lower extremities. Four trials included patients with venous ulcers, one trial with arterial ulcers, and one with ulcers of mixed causes. Serum zinc was measured in four trials, and oral zinc sulfate was compared with a placebo in patients with venous ulcers in other trials. Overall, there was no evidence that oral zinc promotes the healing of leg arterial or venous ulcers, and the sample size was insufficient to rule out this possibility.

## 4. Endocrine and metabolic diseases

### 4.1. Acrodermatitis enteropathica

Acrodermatitis enteropathica (AE) is a type of zinc deficiency that is more common in infants and young children. Hereditary AE is caused by gene mutations and is inherited as an autosomal recessive disorder. Acquired AE results from zinc deficiency due to various acquired causes. The typical symptoms of AE are periorificial and acral dermatitis, diarrhea, and hair loss. Kury et al. (89) identified SLC39A4 as one of the causative genes of hereditary AE. Mutations in the SLC39A4 gene resulted in decreased plasma membrane zinc transporters and defective degradation of the extracellular amino terminus of ZIP4, which caused a decrease in the intestinal uptake of zinc (90). Therefore, zinc supplementation is an effective means of treating AE. ZnSO4 (3 mg/kg/day) is recommended for hereditary AE. Complete resolution of clinical symptoms occurs within 2–4 weeks, after which maintenance doses (1–2 mg/kg/day) may be used. Regarding acquired AE, the recommended dose is 0.5–1 mg/kg/day for children and 15–30 mg/kg/day for adults (91). Serum zinc levels need to be measured during the treatment of AE and, more importantly, to identify specific treatments for the cause.

### 4.2. Necrolytic migratory erythema

Necrolytic migratory erythema (NEM) is a rare inflammatory skin reaction. It is the most common skin manifestation in patients with glucagonoma. Its clinical manifestations are recurrent migratory necrolysis ring-shaped or dark gyrus erythema, extensive scab, scale and discharge, and stomatitis. However, this may be seen even in patients with normal or elevated glucagon levels who do not have glucagonoma. Ferrara et al. (92) summarized a range of NMEs caused by factors other than glucagonoma. These include pancreas disorders other than glucagonoma, liver diseases, bowel diseases, neuroendocrine tumors other than glucagonoma, and malabsorption. NME can also be called acquired acrodermatitis enteropathica when it is connected with poor intestinal absorption and deficiencies of necessary amino acids and minerals. Causes of NME include glucagon-induced cutaneous necrolysis, hypoaminoacidemia-induced epidermal protein deficiency and necrolysis, dietary or metabolic zinc or essential fatty acids deficiency, glucagon-induced inflammatory mediators, and a wide range of malabsorption (93). Pakran et al. (36) reported a 53-year-old female patient with NME. Her skin lesions responded to oral zinc sulfate and monthly octreotide injections. A case report by Salaheldin et al. (94) showed NME because of serious zinc deficiency since modified Roux-en-Y gastric bypass surgery. Therefore, they considered NME a severe and rare dermatological complication of serious zinc deficiency. Based on a review of 13 studies, Salaheldin et al. (94) identified that obesity was a risk factor for zinc deficiency. Failure to follow diet or vitamin management, postoperative complications leading to vomiting or diarrhea, adverse follow-up care, and poor absorption may induce or aggravate the original zinc deficiency. It is recommended to pay attention to the nutritional indicators of patients before and after bariatric surgery and to receive nutritional supplements immediately if nutritional deficiencies are found.

### 4.3. Necrolytic acral erythema

Necrolytic acral erythema (NAE) is a cutaneous manifestation of dark erythema patches with blisters and hyperkeratosis occurring on the dorsal side of the acral (95). It was first described by el Darouti and Abu el Ela in 1996 (96). It used to be considered a characteristic manifestation of active HCV infection. However, several seronegative cases have been reported in recent years (97–99). Moneib et al. (100) compared zinc levels in serum, lesion, and perilesional skin from 15 NAE patients and 10 healthy patients (patients group: serum 0.44 ± 0.13 mg/L, lesion 42.6 ± 18.9 mg/L, perilesional skin 32.5 ± 17.2 mg/L; control group: serum 1.17± 0.29 mg/L, skin 100.1± 2.77 mg/L). The results displayed that serum zinc levels were significantly lower in patients with a positive correlation between lesional and perilesional skin zinc (r = 0.91, *P* < 0.01). However, their studied patients were HCV-positive. Pandit et al. (99) reported two HCV-seronegative NAE cases with low serum zinc levels (case 1: 54.56 μg/dL, reference range: 70–120 μg/dL; case 2 was not mentioned). Oral zinc therapy is remarkably effective. Abdallah et al. (37) reported a case of an HCV-positive but average serum zinc level in a patient with NAE. The patient was given oral zinc sulfate 220 mg twice a day. The skin lesions gradually subsided within 8 weeks until the symptoms completely subsided and histological examination showed no apparent abnormalities. The patient reduced the dose of zinc sulfate to once daily, and signs and symptoms worsened. Zinc may play a causative role, given the responses to supplementation reported in the literature (38, 39). Zinc deficiency is considered one of the possible causes of the pathogenesis of NAE. In patients with normal serum zinc levels, it has been suggested that reduced serum zinc levels are a late manifestation of zinc deficiency and that skin manifestations in such patients may already present with zinc deficiency.

## 5. Disorders of hair

### 5.1. Alopecias

Zn is involved in important functional activities in the hair follicles. It potently inhibits hair follicle regression and accelerates hair follicle recovery. Androgenetic alopecia (AGA), alopecia areata (AA), and telogen effluvium (TE) are common hair loss problems. Concentrations of serum zinc and copper were measured in 30 healthy volunteers and 312 patients with AA, male alopecia, female alopecia, and TE. Mean serum zinc levels of all types of alopecia patients (84.33 ± 22.88 μg/dL) were significantly lower than those of the controls (97.94 ± 21.05 μg/dL) (*P* = 0.002), especially the AA group and the TE group. However, serum copper levels were not significantly different among the groups (101). Chang et al. (102) assessed the association between serum zinc levels and male AGA. Dhaher et al. (103) estimated serum and hair zinc (65.6 ± 14.2 vs. 128.4 ± 41.4 μg/dL for serum and 103.4 ± 25.5 vs. 143.5 ± 33.1 ppm for hair) and iron levels in women with AGA. Thompson et al. (104) examined the link between zinc levels and alopecia areata. All the results suggested a decrease in zinc content. Camacho et al. (40) found oral zinc aspartate, biotin, and clobetasol propionate to be good side-effect-free treatment regimens for pediatric patients with AA. Berger et al. (41) compared the impacts of topical 5% minoxidil solution, 1% pyrithione zinc shampoo, and a mixture of the two on the density of hair. A randomized, investigator-blinded, parallel-group clinical trial was conducted on 200 patients exhibiting Hamilton-Norwood type III vertex or type IV baldness over 6 months. Patient and investigator evaluations of overall improvement generally showed a 5% efficacy of minoxidil. The effect of 1% zinc pyrithione shampoo alone is not apparent. Park et al. (42) conducted a 12-week study of the effect of oral zinc supplementation in AA patients with low serum zinc levels. A positive treatment effect was observed in 66.7% of patients (9 of 15), but this was not statistically significant. The serum zinc content of the positive group was significantly higher than that of the opposing group, which may be one of the reasons. Rossi et al. (43) performed an *in vivo* study. Forty patients with AGA and TE were divided into two groups. One group received a combination of zinc and arginine lotion, while the other received a placebo. The treatment lasted for 23 consecutive weeks. Evaluation of all objective parameters showed that the zinc-arginine combination group was more effective than the placebo group.

## 6. Pigmental diseases

### 6.1. Vitiligo

Vitiligo is a primary, localized, or generalized depigmentation of the skin and mucosa. Several studies have found that zinc levels in patients with vitiligo are lower than in controls (105–108). There are very few studies on the role of zinc in therapy, and only Yahoobi et al. reported a clinical trial comparing the therapeutic effect of topical corticosteroids to a combination of oral zinc sulfate and topical corticosteroids. However, the results found no significant difference between the two treatments (44). The adipokine zinc-α2 glycoprotein plays a role in metabolism, melanin, and immune regulation. It is considered one of the participants in the complex pathogenesis of vitiligo (109, 110).

### 6.2. Melasma

Melasma is a chronic skin condition involving excessive production of melanin in areas exposed to ultraviolet radiation (111). The treatment and management of melasma are challenging, with a high recurrence rate and a significant impact on quality of life. The triple combination of 4% hydroquinone, 0.05% retinoic acid, and 0.01% fluocinolone acetonide remains the only US FDA-approved treatment for melasma and has proven to be the gold standard among races (112). Because of zinc's peeling, antioxidant, anti-inflammatory, and astringent properties and its proven therapeutic benefits in other skin diseases, such as Leishmania, Sharquie et al. (45) treated 28 patients of melasma with a 10% zinc sulfate solution. A total of 14 patients who completed the trial had a significant decrease in MASI scores, so the authors concluded that the regimen was effective. In this trial, patients were told to wear sunscreen, so it is uncertain whether sunscreen affected the results, which is one of the questions Miot et al. (113) raised in their article. Iraji et al. (46) and Yousefi et al. (47) compared zinc sulfate with hydroquinone to assess the efficacy of topical zinc application. Both studies concluded that zinc was less effective than hydroquinone in reducing melasma severity. A double-blind randomization with a sufficient sample size would have given us better guidance.

## 7. Premalignant and malignant diseases

### 7.1. Basal cell carcinoma

Basal cell carcinoma (BCC) is one of the most common skin malignancies, and its pathogenesis is closely related to long-term sun exposure. BCC usually occurs in the exposed parts of elderly individuals, especially the face. As a sunscreen component, zinc oxide can prevent BCC by effectively preventing photodamage. Majidi et al. (114) revealed an evident decrease in serum zinc levels of patients with BCC compared to the controls (78.65 ± 12.83 vs. 89.39 ± 12.47 μg/dL, *P* = 0.000), probably because zinc was consumed during oxidative stress. Immunohistochemical staining of skin cancer biopsies demonstrated reduced levels of the antioxidant enzymes catalase and copper-zinc superoxide dismutase in BCCs and their surrounding tissues compared with young control skin (115). In addition to preventing BCC, zinc may also be used in treatment. In an open-label case interventional study, 100 BCC lesions were significantly improved with intralesional 2% zinc gluconate solution with no apparent side effects (All lesions showed clinical cure: 18 (18%) lesions after the first injection, 52 (52%) lesions after second injections, 29 (29%) lesions after third injections and one (1%) lesion after the fourth injection) (48). There is little literature on treating BCC with zinc, and further research is needed.

### 7.2. Actinic keratosis

Actinic keratosis (AK) is a precancerous lesion caused by long-term sun exposure. The disease can develop into non-melanocytic skin tumors without treatment. Similar to BCC, zinc oxide is also effective in preventing AK. A single-blind therapeutic trial showed that applying 25% zinc sulfate to the lesion site twice daily significantly heals the lesion (49). Zinc has the functions of anti-oxidation, sun protection, enhancing DNA repairs, boosting the immune system, and accelerating the apoptosis of malignant cells, which is beneficial to AK therapy.

## 8. Miscellaneous dermatoses

### 8.1. Bullous skin diseases

Bullous skin diseases are characterized by bullae, some of which were revealed to be related to zinc. A study reported that the mean serum zinc and copper levels and copper/zinc ratio were significantly lower in Iranian patients with pemphigus vulgaris than in controls (serum zinc: 914.2 ± 311.9 vs. 1,207.2 ± 173.8 μg/L, *P* < 0.01; serum copper: 769.6 ± 240.7 μg/L vs. 1,642.8 ± 334.4 μg/L, *P* < 0.01; Cu/Zn: 1.2 ± 1.6 vs. 1.4 ± 0.3, *P* < 0.01) (91). The possible mechanism of the decrease in serum zinc and copper in pemphigus vulgaris needs to be further explored. In a case report, Calogero et al. (50) observed that using 50% zinc oxide paste alone ultimately improved the lesion of Hailey-Hailey disease (HHD); however, recovery was delayed with tacrolimus and 50% ZnO. This was a side-by-side comparison and may provide hints for the treatment of HHD.

### 8.2. Erosive pustular dermatosis

Erosive pustular dermatosis (EPD) is a rare inflammatory skin disease. The disease is characterized by sterile pustules with keratotic or erosive plaques, typically involving the scalp or legs. Typically, EPD occurs on legs with venous insufficiency or atrophic skin caused by actinic damage on the scalp (116). The literature has described many treatments. However, there is no agreement on the optimal treatment for EPD. According to research, topical corticosteroids are the most frequently used treatment for EPD. Lee et al. (117) systematically evaluated the literature on various treatments and their efficacy in EPD management. They summarized six studies on the use of zinc derivatives as a treatment that can bring relief to patients. Junejo et al. (118) performed a systematic review of erosive pustular dermatosis of the scalp. The results showed that the limited evidence supports the use of topical corticosteroids, with or without oral zinc, followed by maintenance therapy with topical calcineurin inhibitors as effective in managing this condition. However, Di Altobrando et al. (51) found that topical zinc oxide may be a unitary, effective, and inexpensive strategy for the chronic therapy of EPD of the legs. The efficacy of zinc oxide in erosive pustular dermatosis of the scalp was also validated in a later study by Di Altobrando et al. (52).

## 9. Conclusion

Zinc is a trace element necessary to maintain the physiological functions of the skin. Impaired zinc signaling is related with abnormal zinc status, thereby causing specific pathophysiological conditions associated with the disease. The analysis of serum zinc and hair zinc detection results in patients with warts, hidradenitis suppurativa, cutaneous leishmaniasis, acne, eczema, psoriasis, hair loss, necrolytic migratory erythema, and so on, we observed a decrease in zinc levels in all of these diseases. These measured abnormalities in zinc levels may be related to the pathogenesis of the skin disorders mentioned above.

This study provides a reference and basis for treating dermatosis to supplement zinc preparations. Zinc preparations commonly used by researchers include zinc sulfate, zinc acetate, zinc gluconate, and zinc pyrithione. The dosage of the drug and the route of administration varied from study to study. Zinc preparations can be used in a variety of ways, such as external application, oral administration, local injection, and even as clothing. These methods are simple and easy to perform and can ensure patient compliance. Among the many experiments, zinc sulfate is the most commonly used. Its efficacy in treating acrodermatitis enteropathica, warts, acne, ulcers, and cutaneous leishmania is well-studied. Zinc sulfate has higher zinc content and absorption compared with other zinc preparations, which may be a good choice for future clinical research. The effective zinc dose also needs to be reported in these trials (119).

The role of zinc supplementation in acrodermatitis enteropathies is not in doubt. In addition, zinc's effect in treating viral warts, genital herpes, cutaneous leishmaniasis, ulcers, and wounds is significant, and there is evidence from randomized controlled trials. Momen-Heravi et al. (34) found a significant rise in serum zinc levels in the treatment group. Most studies did not measure changes in zinc levels before and after treatment interventions. The relationship between zinc levels and skin symptoms deserves attention. The sample size of the above trials could be further expanded. In summary, for these skin diseases, zinc can be used as an adjuvant therapy or as a choice when traditional treatment is ineffective, with considerable promise.

Sharquie et al. (22) found that zinc sulfate was effective in the treatment of rosacea, whereas Sharquie et al. (23) showed no difference between zinc sulfate treated and control groups. The dose and frequency of zinc used in the two trials differed slightly. Topical zinc sulfate improved MASI scores in melasma. However, Iraji et al. (46) and Yousefi et al. (47) found the decrease in MASI scores was more pronounced in the control group. Yahoobi et al. (44) reported that there was no significant difference between topical corticosteroids and a combination of oral zinc sulfate and topical corticosteroids for the treatment of vitiligo. Therefore, the efficacy of zinc in these diseases needs to be further studied and should be applied with caution.

As for other skin disorders, like necrolytic acral erythema, oral lichen planus, leprosy, and Hailey-hailey disease, case reports or trials with small samples often dominate and the evidence is weak. Because of the lack of more systematic trial studies, zinc treatment can only be temporarily used as a possible option.

In the future, in addition to more clinical studies of zinc in the treatment of skin disorders, more information about the pathophysiological processes caused by zinc deficiency and the application of zinc [like zinc oxide-impregnated fabrics (25) and zinc oxide tetrapod nanoparticles (60)] are worth looking forward to.

## Author contributions

CY and YC designed the study. PZ and YD managed, analyzed the data, prepared the first draft, and reviewed and edited the manuscript, with comments from CY and YC. All authors were involved in revising the paper, had full access to the data, and gave final approval of the submitted versions.
